# Microscopic ligation combined with shunt procedure versus microsurgical varicocelectomy for nutcracker syndrome and left varicocele: a randomized controlled trial

**DOI:** 10.1186/s12610-026-00312-6

**Published:** 2026-04-17

**Authors:** Ke Dou, Xinchen Zeng, Qi Yang, Yutao Li, Lei Wang, Tao Song, Juncheng Yao

**Affiliations:** 1 Department of Urology, Sichuan Provincial People’s Hospital, University of Electronic Science and Technology of China, Chengdu, 610041 Sichuan China; 2 Reproductive Medicine Center, Mianyang Central Hospital, School of Medicine, University of Electronic Science and Technology of China, Mianyang, 621000 Sichuan China; 3 Department of Assisted Reproduction Center, Sichuan Provincial People’s Hospital, University of Electronic Science and Technology of China, No.32, Section 2, West 1 Ring Road, Qingyang District, Chengdu, 610041 Sichuan China; 4 Department of Reproduction and Infertility, Chengdu Women’s and Children’s Central Hospital, School of Medicine, University of Electronic Science and Technology of China, Chengdu, 610073 China

**Keywords:** Nutcracker syndrome, Varicocele, Microsurgery, Anastomosis, Semen quality, Renal vein, Syndrome du casse-noisette, Varicocèle, Microchirurgie, Anastomose, Qualité du sperme, Veine rénale

## Abstract

**Background:**

This study is to evaluate the safety and efficacy of a novel microsurgical approach (Microsurgical Spermatico-Inferior Epigastric Vein Anastomosis with Selective Venous Disconnection, MSEA-SVD) compared to conventional Microsurgical Varicocelectomy approach in patients with Nutcracker Syndrome and left-sided varicocele. This single-center, randomized controlled trial enrolled 50 patients (25 per group) diagnosed with Nutcracker Syndrome and left-sided varicocele. Group A underwent MSEA-SVD, while Group B received Microsurgical Varicocelectomy. The primary outcomes included semen quality, renal vein hemodynamics, and varicocele recurrence. Follow-up assessments were conducted at 1, 3, and 6 months postoperatively using color Doppler ultrasound and semen analysis. Statistical comparisons were performed using IBM SPSS 27.0.

**Results:**

MSEA-SVD achieves superior outcomes in semen quality and renal vein decompression compared to Microsurgical Varicocelectomy but requires a longer surgery time and hospitalization.

**Conclusions:**

With careful patient selection, MSEA-SVD may become a preferred approach for managing Nutcracker Syndrome-related varicocele.

## Résumé

**Contexte:**

Cette étude visait à évaluer la sécurité et l’efficacité d’une nouvelle approche microchirurgicale (Anastomose microchirurgicale spermatico-inférieure de la veine épigastrique avec déconnexion veineuse sélective, MSEA-SVD) par rapport à l’approche microchirurgicale conventionnelle de la varicocèlectomie chez les patients atteints du syndrome de Casse-Noisette et de varicocèle du côté gauche. Cet essai contrôlé randomisé et monocentrique a recruté 50 patients (25 par groupe) diagnostiqués avec un syndrome du Casse-Noisette et une varicocèle du côté gauche. Le groupe A a subi un MSEA-SVD, tandis que le groupe B a eu une varicolectomie microchirurgicale. Les objectifs principaux comprenaient la qualité du sperme, l’hémodynamique des veines rénales et la récidive du varicocèle. Les évaluations de suivi ont été réalisées à 1, 3 et 6 mois après l’opération à l’aide d’échographies Doppler couleur et d’une analyse sperme.

**Résultats:**

La MSEA-SVD a obtenu des résultats supérieurs de la qualité du sperme ainsi qu’une meilleure décompression des veines rénales comparés à ceux de la varicolectomie microchirurgicale, mais nécessitant une durée chirurgicale et une hospitalisation plus longues.

**Conclusions:**

Avec un choix attentif des patients, la MSEA-SVD pourrait devenir une approche privilégiée pour gérer la varicocèle liée au syndrome du Casse-Noisette.

## Introduction

 The Nutcracker Syndrome (NCS), clinically termed Left Renal Vein Entrapment Syndrome, refers to a vascular compression disorder characterized by hematuria, proteinuria, varicocele, and pelvic congestion symptoms resulting from anatomical compression of the left renal vein between the abdominal aorta and superior mesenteric artery [1]. First identified by El-Sadr and Mina in their landmark cadaveric study [2], the condition was later formally named “Nutcracker Syndrome” by De Schepper through angiographic documentation [3]. Since then, medical interest in NCS has steadily grown, leading to an evolving understanding of its pathophysiology. Although historically considered rare, improved diagnostic modalities including computed tomography angiography have increased detection rates by 38% over the past decade [4–5]. Epidemiological data suggest a bimodal age distribution, with peak incidence occurring in slender individuals aged 18–40 years. Nevertheless, significant knowledge gaps persist regarding its true population prevalence, and a definitive epidemiological report on NCS is still lacking [6–7].

The therapeutic paradigm for NCS remains controversial due to heterogeneous clinical presentations and natural history [8]. In pediatric patients, vascular compression may be relieved naturally as retroperitoneal fat pad develops with growth and weight gain. Therefore, first-line management emphasizes conservative strategies, with pharmacological adjuncts such as angiotensin-converting enzyme inhibitors (ACEIs) and antiplatelet agents to address hematuria and prevent microthrombosis [1, 9]. Some scholars recommend delaying surgical intervention untilsymptoms persist beyond six months of conservative management in adults or remain refractory to 24-month observation in pediateric patients [10]. Historically, earlier approaches included open surgery, laparoscopic, robot-assisted transposition of the superior mesenteric artery, left renal vein transposition, and autologous kidney transplantation. However, these methods have largely been phased out due to high complication rates [11–13]. Historically, earlier approaches included open surgery, laparoscopic, robot-assisted transposition of the superior mesenteric artery, left renal vein transposition, and autologous kidney transplantation. However, these methods have largely been phased out due to high complication rates [10, 14–16].

Varicocele (VC) is a common vascular disorder of the male urogenital system, characterized by abnormal dilation or tortuosity of the pampiniform plexus within the scrotum. VC affects approximately 10–15% of the general male population, but it rises significantly in men experiencing infertility, reaching rates of 50–80% [17–18]. The pathophysiological triad of VC consists of three key mechanisms: (i) thermal stress, where scrotal temperature elevation (≥ 1.5 °C) impairs spermatogenesis (ii) venous hypertension, where increased testicular interstitial pressure (> 15 mmHg) disrupts the blood-testis barrier and (iii) neurohormonal reflux, where retrograde transport of adrenal-derived 8-OHdG and noradrenaline induces oxidative DNA damage [19–20]. Chronic exposure (> 24 months) to this microenvironment reduces total motile sperm count and increases sperm DNA fragmentation index [20].

The therapeutic landscape for varicocele management continues to evolve, with ongoing debate regarding surgical indications [21]. Current consensus recommends conservative management with regular follow-up for asymptomatic patients with subclinical or Clinical Grade I presentations [22]. Past surgical options included embolization of the spermatic vein and high ligation of the spermatic vein under laparoscopy. However, with advances in microsurgical techniques, microscopic varicocelectomy has become the preferred method. Compared to laparoscopic varicocelectomy, microscopic varicocelectomy better protects the arterial and lymphatic structures of the spermatic cord, promoting improved postoperative recovery [23].

The relationship between NCS and VC has historically been underrecognized in clinical practice, with both conditions often regarded as independent. However, when NCS manifests solely as VC, this oversight can lead to suboptimal management. Patients may undergo immediate microscopic simple ligation based on an initial diagnosis of VC. Yet, despite surgery, recurrence of VC often occurs, prompting clinicians to then consider the possibility that VC may have been caused by NCS. This oversight arises because the pathophysiological interplay between NCS and left-sided varicocele (LVC) is not always recognized, few urologists routinely assess renal venous hemodynamics in LVC patients, leading to frequent recurrence [24]. Therefore, for patients diagnosed with Grade II or higher VC, evaluating for left renal vein compression is recommended .

To address this therapeutic gap, we developed a hemodynamic-guided microsurgical approach integrating two key components: Microsurgical Spermatico- Inferior Epigastric vein Anastomosis(MESA): which establishes collateral drainage via the inferior epigastric vein, Selective Venous Disconnection(SVD), which reserves 2–3 lymphatic channels and the cremasteric artery. This study aims to evaluate the safety and early-to-mid-term efficacy of this surgical approach and compare its outcomes with those of patients undergoing microsurgical varicocelectomy(MSV) .

## Patients and methods

### Study design

**Fig. 1:**
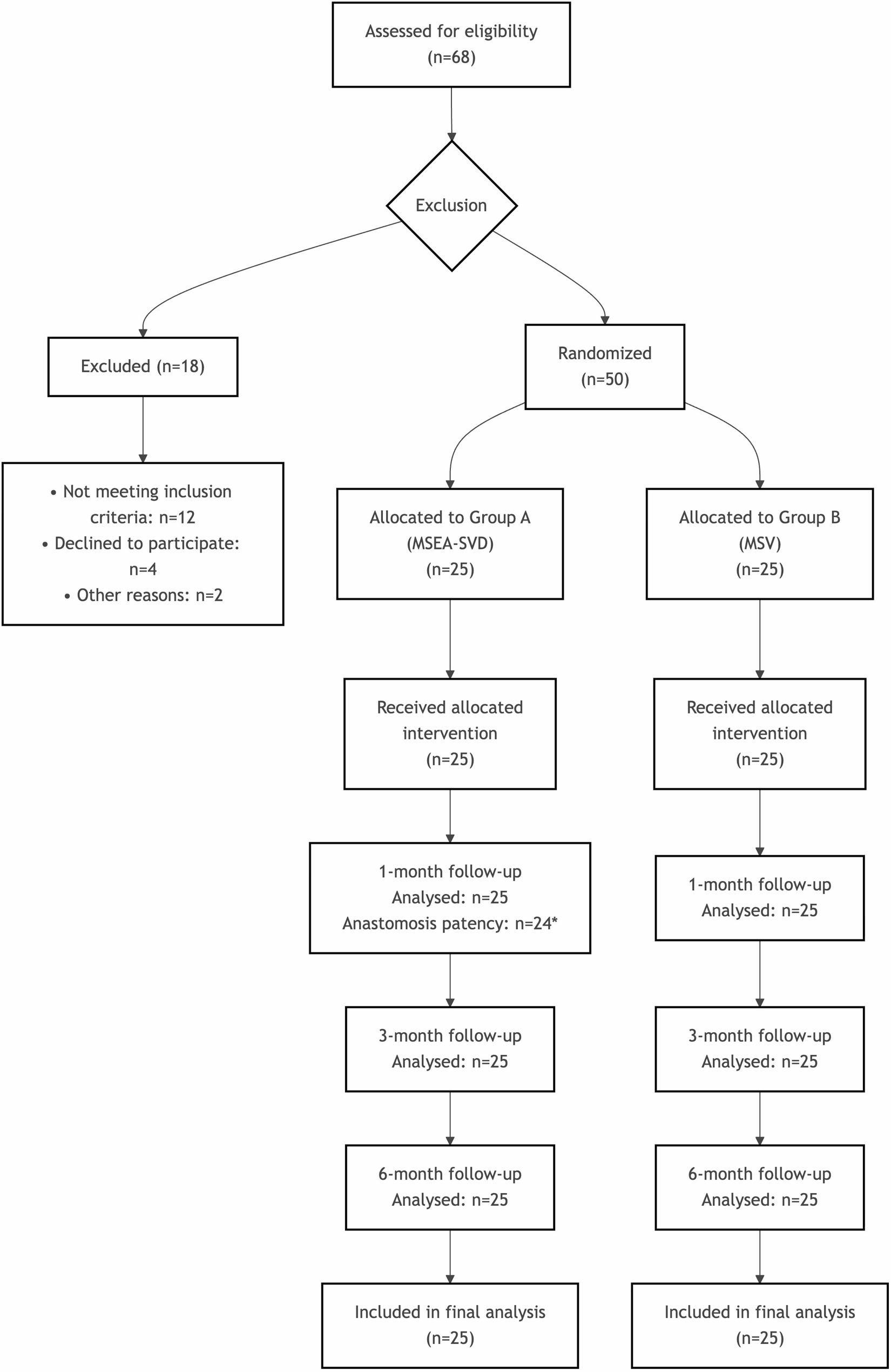
Flowchart of patient recruitment, randomization, and follow-up

This was a single-center, randomized controlled trial conducted at the Department of Urology, Sichuan Provincial People’s Hospital. The study was designed to compare the safety and efficacy of Microsurgical Spermatico-Inferior Epigastric Vein Anastomosis with Selective Venous Disconnection (MSEA-SVD) versus Microsurgical Varicocelectomy (MSV) in patients with Nutcracker Syndrome (NCS) and left-sided varicocele (LVC). The study protocol was approved by the institutional ethics committee, and written informed consent was obtained from all participants (Fig. 1).

### Randomization

Patients who met the inclusion criteria were randomly assigned in a 1:1 ratio to either Group A (MSEA-SVD) or Group B (MSV) using a computer-generated random number sequence. The allocation sequence was concealed using sequentially numbered, opaque, sealed envelopes, which were opened only after the patient provided written informed consent and on the day of surgery. The surgeon performing the operation was not blinded to the group assignment due to the inherent differences in the surgical techniques.

### Blinding

Due to the distinct nature of the two surgical procedures (involving different incision sites and the presence of a vascular anastomosis in Group A), it was not feasible to blind the operating surgeons or the patients to the treatment allocation. However, to minimize assessment bias, the outcome assessors were blinded to the group assignment. Specifically, the ultrasound physicians who performed all postoperative scrotal and renal vein Doppler ultrasound examinations, and the laboratory technicians who conducted the semen analyses, were not informed of the patients’ surgical group allocation throughout the entire follow-up period.

### Patients

Fifty patients with NCS and LVC were screened based on predefined inclusion and exclusion criteria. Inclusion Criteria: (a) Patients admitted to the Department of Urology and Andrology at Sichuan Provincial People’s Hospital between July 2021 and June 2023 for surgical treatment; (b) Meeting the diagnostic criteria for Nutcracker Syndrome and varicocele [1, 22, 25]: ①Color Doppler ultrasonography of the left renal vein in the supine position demonstrates a ratio of peak flow velocity at the aortomesenteric angle to the flow velocity at the renal hilum greater than 5; ②Computed tomography venography (CTV) reveals a ratio of the internal diameter at the widest portion of the left renal vein near the renal hilum to that at the narrowest portion within the aortomesenteric angle greater than 4, accompanied by an angle of less than 40 degrees; ③Physical examination reveals palpably dilated spermatic veins or visible tortuous, dilated vascular clusters, often described as a “bag of worms,” in the scrotal region; ④Scrotal color Doppler ultrasonography performed in the supine position during quiet respiration shows a spermatic vein internal diameter greater than 2.2 mm, with reflux lasting more than 2 s during the Valsalva maneuver); ⑤Aged 18–45 years; ⑥Normal preoperative findings on chest X-ray, electrocardiogram, and laboratory tests of renal function, liver function, and coagulation profile; ⑦Completion of postoperative follow-up assessments at 1, 3, and 6 months after surgery, including clinical examinations in the Urology and Andrology outpatient clinic, as well as relevant auxiliary investigations performed in the Reproductive Medicine Center, Ultrasound Department, and Clinical Laboratory. Exclusion Criteria: (a) Previous surgical intervention for Nutcracker Syndrome or varicocele; (b) Secondary varicocele caused by retroperitoneal tumors, renal or inferior vena cava tumor thrombi, etc.; (c) Concurrent conditions that may interfere with the evaluation of surgical outcomes, such as spinal deformities, urinary tuberculosis, urolithiasis, tumors, glomerulonephritis, autoimmune diseases, phlebitis, or lower extremity varicose veins; (d) Diseases affecting outcome measures, including azoospermia, severe oligoasthenoteratozoospermia, chromosomal abnormalities, or hormonal disorders; (e) Presence of bilateral varicocele; (f) Comorbid severe systemic conditions such as hypertension or diabetes mellitus; (g)Incomplete preoperative or postoperative data; (h) Evidence of collateral venous drainage pathways in the left renal vein as indicated by CT venography.

All procedures were performed under the lead superviion of a single experienced surgeon. Patients were divided into two groups (*n* = 25 per group) according to the surgical method: Group A underwent MSEA-SVD, while Group B received MSV. Preoperative clinical manifestations and examination results were recorded, and postoperative evaluations were conducted at 1, 3, and 6 months. Outcome measures included recurrence rate, symptom improvement, imaging findings, semen parameter recovery, and other relevant indicators.

### Surgical techniques

#### Microsurgical subinguinal varicocelectomy(MSV)

Patients in Group B underwent the MSV procedure. After thorough preoperative preparation and induction of general anesthesia, a 1.5-cm incision was made just below the left inguinal external ring, oriented perpendicular to the course of the spermatic cord. The skin, subcutaneous fat, Camper’s fascia, and Scarpa’s fascia were sequentially incised and separated. Once the spermatic cord was exposed, it was gently elevated using a appendix forceps and retracted with a soft plastic sling to facilitate further dissection. The external spermatic fascia and the cremaster muscle were then carefully incised and separated to fully expose the underlying cord structures. Under 5–10× magnification with an operating microscope (Carl Zeiss OPMI VARIO 700, Germany), the arterial, venous, lymphatic, and neural structures within the cord were meticulously identified. Each spermatic vein was double ligated individually to ensure complete occlusion, while all adjacent arteries, nerves, and lymphatic vessels were preserved. Finally, the incision was closed in layers with (3 − 0) absorbable sutures, restoring the anatomical integrity of the region.

#### Microsurgical spermatico- inferior epigastric vein anastomosis and selective venous disconnection (MESA-SVD)

**Fig. 2:**
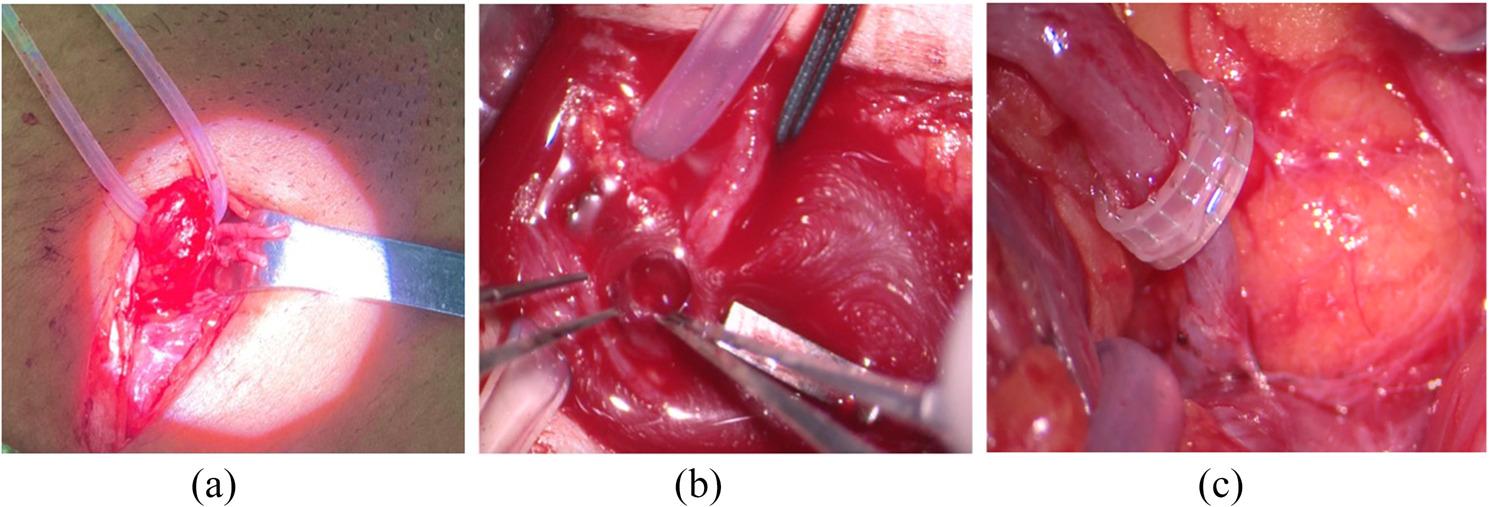
Intraoperative pictures of MSEA-SVD: **a** Separating the proximal end of the spare deep inferior epigastric vein; **b** Obvious proximal spermatic vein reflux was found during operation; **c** The proximal end of the spermatic vein and the proximal end of the deep inferior epigastric vein were anastomosed end-to-end using a vascular stapler, with the spermatic vein above the stapler and the deep inferior epigastric vein below the stapler.

Group A patients underwent a combined microsurgical vein anastomosis procedure. After full preoperative preparation and induction of general anesthesia, key anatomical landmarks—including the inferior epigastric vein, spermatic cord, and the inguinal ligament—were precisely identified and marked using a sterile surgical marker. A 3-cm oblique incision was made in the left inguinal region. Camper’s fascia and Scarpa’s fascia were carefully dissected to expose the spermatic cord. Under an operating microscope set at 5–10× magnification (Carl Zeiss OPMI VARIO 700, Germany), the inferior epigastric artery and its accompanying veins were located. As shown in Fig. 2(a), at least 4–5 cm of the inferior epigastric vein was preserved to ensure a tension-free anastomosis. As can be seen from Fig. 2(b), the proximal end of the inferior epigastric vein was flushed with heparinized saline to observe venous return and assess valve function, while the distal end was doubly ligated using 4 − 0 silk sutures. If these conditions were met, under 8–10× surgical magnification, the proximal ends of the spermatic vein and the inferior epigastric vein were anastomosed in an end-to-end fashion using 8 − 0 nonabsorbable sutures or a vascular anastomosis device (the anastomosis method is selected according to the patient’s vascular condition and preference), as As indicated in Fig. 2(c). Postoperatively, patients received prophylactic heparin and aspirin to prevent thrombosis, thereby ensuring optimal patency of the anastomosis.

### Semen analysis

Semen samples were collected by masturbation after a recommended 2–7 days of sexual abstinence. Semen analysis was performed in accordance with the standard protocols outlined in the World Health Organization Laboratory Manual for the Examination and Processing of Human Semen (5th edition). All analyses were performed manually by experienced technicians. Sperm concentration and motility (including progressive motility-PR) were assessed using a Makler counting chamber. Sperm morphology was evaluated after Diff-Quik staining, and classification followed the strict Tygerberg criteria as defined in the WHO manual (5th edition). The andrology laboratory participates in internal quality control programs, and technicians undergo regular competency assessments to ensure the consistency and reliability of results.

### Statistical analysis

Clinical data were analyzed using IBM SPSS Statistics 27.0. Categorical variables were compared using Pearson’s chi-square test, while continuous variables were expressed as mean ± standard deviation (SD). For normally distributed data, independent-sample t-tests were used for inter-group comparisons; for non-parametric data, Mann-Whitney U tests were applied.Paired comparisons were conducted with Wilcoxon signed-rank tests. All statistical were two-tailed, with a P value < 0.05 considered statistically significant.

### Sample size calculation

Although this was an initial randomized controlled trial without a prior formal sample size calculation, a post-hoc power analysis was conducted using G*Power software (version 3.1.9.7) to assess the statistical power of our findings. The primary outcome for this analysis was the difference in sperm progressive motility (PR) at 6 months postoperatively between the two groups. With an effect size (d) of 1.02, derived from the observed group means and standard deviations (Group A: 38.24 ± 6.54%; Group B: 29.36 ± 10.60%), a sample size of 25 per group, and an alpha level of 0.05, the achieved statistical power (1-β) for a two-tailed independent t-test was calculated to be 0.96 (96%). This indicates that the study had a high probability of detecting a statistically significant difference in the primary outcome, should one exist, thus strengthening the validity of the conclusions drawn from the observed results.

## Results

### Preoperative comparison between groups

Preoperative indicators—including age, BMI, serum hormone levels, semen quality, and imaging findings—were statistically analyzed for both groups. As shown in Table 1, there were no significant differences between Group A and Group B across these parameters(all *P* > 0.05), confirming comparability for subsequent postoperative analysis. Furthermore, as detailed in Table 2, there were no statistically significant differences in the distribution of preoperative clinical manifestations between Group A and Group B. The majority of patients presented with scrotal discomfort (62%), infertility (52%), and semen abnormalities, particularly asthenospermia (70%) and teratospermia (82%). Most patients had Clinical Grade III varicocele (84%). The comparable distribution of these clinical features between groups further supports the baseline homogeneity of the study population.

**Table 1:** Clinical characteristics of patients

Characteristic	Group A *n*=25	Group B *n*=25	*P *Value
Demographics			
Age（years）	25.72±5.22	26.08±4.70	0.799
BMI（kg/m^2^）	19.88±2.54	19.43±2.33	0.515
Serum hormone Levels			
T（nmol/L）	14.95±3.39	14.07±3.44	0.367
LH（IU/L）	6.84±2.84	5.76±2.84	0.248
FSH（IU/L）	5.43±1.98	4.69±1.90	0.188
INH B（pg/mL）	187.33±77.80	181.14±94.16	0.961
Semen Quality			
Spermconcentration（×10^6^/ml）	27.77±15.31	28.08±9.37	0.467
PR（％）	26.12±6.09	24.48±11.82	0.087
Normal sperm morphology（％）	2.22±1.12	2.46±1.20	0.428
CTV Parameters			
Angleat compression site（°）	24.90±10.07	24.29±11.37	0.741
Leftrenalveindiameterratio	5.58±1.20	6.14±1.76	0.313
Color Doppler ultrasound results			
Leftspermatic			
veindiameter（mm）	3.60±0.48	3.57±0.50	0.818
reflux time（s）	4.46±1.34	3.92±1.58	0.255
Left testicular volume（ml）	12.26±2.22	11.63±1.29	0.335
Testicular asymmetry percentage（％）	25.70±13.42	21.44±11.35	0.231
Left renal vein peak velocity（PV）			
PV1（cm/s）	133.70±25.37	126.69±17.92	0.479
PV2（cm/s）	19.90±6.60	18.26±3.67	0.287
PVR（PV1/PV2）	7.56±3.19	7.13±1.68	0.712

Data are presented as mean ± SD. Continuous variables were compared using independent-sample t-tests or Mann-Whitney U tests as appropriate.

*Abbreviations*: *BMI* Body mass index, *T* Testosterone, *LH* Luteinizing hormone, *FSH* Follicle-stimulating hormone, *INH B* Inhibin B, *PR* Progressive motility, *CTV* Computed tomography venography, *PV1* Blood flow velocity at the compressed site of the left renal vein, *PV2* Blood flow velocity at the widest part of the left renal vein near the renal hilum, *PVR* Left renal vein blood flow velocity ratio (PV1/PV2)

**Table 2:** Comparison of preoperative clinical manifestations between groups

Clinical Manifestations	Group A *n*=25	Group B *n*=25	*P* Value
Scrotal dragging sensation	14	17	0.382
Low back pain	9	8	0.765
Urinary abnormalities			
Hematuria	6	4	0.480
Proteinuria	6	4	0.480
Infertility	15	11	0.258
Semen abnormalities			
Oligospermia	5	2	0.221
Asthenospermia	15	20	0.123
Teratospermia	22	19	0.269
Varicocele grade			0.264
Clinical Grade I	0	1	
Clinical Grade II	2	5	
Clinical Grade III	23	19	

Data are presented as number of patients. Categorical variables were compared using Pearson’s chi-square test or Fisher’s exact test as appropriate. All P values > 0.05 indicate no statistically significant differences in preoperative clinical manifestations between the two groups, confirming baseline comparability

### Comparison of surgical duration and hospital stay (Table 3)

**Table 3:** Comparison of operation duration and hospital stay between groups A and B

		Group A *n* = 25		Group B *n* = 25	*P* Value
Surgical duration (min)	116.08 ± 28.3		50.92 ± 8.02		<0.001
Total hospital stay (days)	6.00 ± 1.68		1.88 ± 0.83		<0.001
Postoperative hospital stay(days)	4.36 ± 1.22		1.28 ± 0.46		<0.001

Data are presented as mean ± SD. Intergroup comparisons were performed using independent-sample t-tests

### Follow-up analysis of anastomosis in group A (Table 4)

**Table 4:** Postoperative anastomosis patency in group A patients

	Inner diameter (mm)	blood flow velocity(cm/s)	
1 month postoperatively(*n* = 24)	1.13 ± 0.22		6.51 ± 4.86
3 month postoperatively (*n* = 25)	1.22 ± 0.25		8.86 ± 2.47
6 month postoperatively (*n* = 25)	1.34 ± 0.31		8.95 ± 2.01

Data are presented as mean ± SD. Intragroup comparisons across time points were performed using repeated measures ANOVA with post-hoc tests. One patient who received vascular anastomosis device had the anastomosis site not visible by color Doppler ultrasound at 1-month follow-up due to scar tissue obscuring the view

In Group A, the proximal ends of the left spermatic vein and the inferior epigastric vein were anastomosed intraoperatively using either an 8 − 0 vascular suture or a vascular anastomosis device, selected based on the vascular condition and patient preference. Color Doppler ultrasound evaluations were performed in the supine position under standardized conditions (room temperature ≈ 25 °C) by the same ultrasound physician at 1, 3, and 6 months postoperatively.

### Postoperative semen quality

Table 5 reveals that no significant postoperative changes were observed in T, FSH, LH, or inhibin B postoperatively in either group.

**Table 5:** Semen parameters at postoperative follow-up time points

	Group A *n* = 25	Group B *n* = 25	*P* Value
Sperm concentration(×10^6^/ml)			
1 month postoperatively	28.62 ± 15.78	26.56 ± 10.84	0.861
3 month postoperatively	32.20 ± 17.43	28.80 ± 8.63	0.907
6 month postoperatively	36.88 ± 12.54	29.32 ± 10.34	0.024
PR(%)			
1 month postoperatively	28.44 ± 7.90	22.08 ± 9.91	0.003
3 month postoperatively	35.16 ± 6.58	24.08 ± 12.27	<0.001
6 month postoperatively	38.24 ± 6.54	29.36 ± 10.60	<0.001
Normal sperm morphology(%)			
1 month postoperatively	2.16 ± 0.86	2.10 ± 0.19	0.761
3 month postoperatively	2.98 ± 0.95	2.28 ± 1.02	0.016
6 month postoperatively	3.26 ± 0.46	2.66 ± 0.86	0.004

Data are presented as mean ± SD. Intergroup comparisons at each time point were performed using independent-sample t-tests. *Abbreviations*: *PR* progressive motility

### Postoperative serum hormone levels (Table 6)

**Table 6:** Serum hormone levels at 6 months postoperatively

	Group A *n* = 25	Group B *n* = 25	*P* Value
T(nmol/L)	14.19 ± 2.65	14.66 ± 3.39	0.596
FSH(IU/L)	6.70 ± 3.26	7.07 ± 3.43	0.594
LH(IU/L)	5.31 ± 1.86	5.45 ± 1.95	0.796
INH B(pg/mL)	173.61 ± 85.84	151.87 ± 72.46	0.338

Data are presented as mean ± SD. Intergroup comparisons were performed using independent-sample t-tests

*Abbreviations*: *T* Testosterone, *FSH* Follicle-stimulating hormone, *LH* Luteinizing hormone, *INH B* Inhibin B

This table reveals that no significant postoperative changes were observed in T, FSH, LH, or INH B postoperatively in either group

### Postoperative scrotal ultrasound findings (Table 7)

**Table 7:** Scrotal Ultrasound Findings at Postoperative Follow-up Time Points

	Group A *n* = 25	Group B *n* = 25	*P* Value
Left spermatic vein diameter(mm)			
1 month postoperatively	1.13 ± 0.55	1.00 ± 0.59	0.488
3 month postoperatively	0.90 ± 0.55	0.88 ± 0.60	0.852
6 month postoperatively	0.69 ± 0.54	0.71 ± 0.73	0.747
Left spermatic vein reflux time(s)			
1 month postoperatively	0.00 ± 0.00	0.00 ± 0.00	/
3 month postoperatively	0.00 ± 0.00	0.00 ± 0.00	/
6 month postoperatively	0.00 ± 0.00	0.00 ± 0.00	/
Testicular asymmetry percentage(%)			
1 month postoperatively	23.78 ± 15.18	19.93 ± 15.58	0.381
3 month postoperatively	18.49 ± 15.17	23.43 ± 12.82	0.220
6 month postoperatively	19.18 ± 13.22	19.12 ± 13.31	0.987

Data are presented as mean ± SD. Intergroup comparisons at each time point were performed using independent-sample t-tests. a. All postoperative reflux times were 0 s in both groups, indicating complete resolution of venous reflux. b. /: Statistical comparison not applicable due to identical values in both groups

### Postoperative left renal vein ultrasound findings (Table 8)

**Table 8:** Left Renal Vein Hemodynamic Parameters at Postoperative Follow-up Time Points

			Group A *n* = 25	Group B *n* = 25	*P* Value
PV1(cm/s)					
1 month postoperatively	83.38 ± 23.96			131.90 ± 19.60	<0.001
3 month postoperatively	65.65 ± 23.77			122.96 ± 12.91	<0.001
6 month postoperatively	46.74 ± 9.78			127.15 ± 17.42	<0.001
PV2(cm/s)					
1 month postoperatively		19.65 ± 5.23		19.94 ± 4.07	0.831
3 month postoperatively		22.68 ± 5.27		21.10 ± 4.85	0.274
6 month postoperatively		18.52 ± 4.91		19.54 ± 4.15	0.430
PVR(PV1/PV2)					
1 month postoperatively		4.54 ± 1.96		6.89 ± 1.71	<0.001
3 month postoperatively		3.08 ± 1.45		6.22 ± 2.00	<0.001
6 month postoperatively		2.61 ± 0.53		6.82 ± 1.84	<0.001

Data are presented as mean ± SD. Intergroup comparisons at each time point were performed using independent-sample t-tests

*Abbreviations*: *PV1* Blood flow velocity at the compressed site of the left renal vein, *PV2* Blood flow velocity at the widest part of the left renal vein near the renal hilum, *PVR* Left renal vein blood flow velocity ratio (PV1/PV2)

## Discussion

As demonstrated in Table 3, group A patients had significantly longer surgical durations, total hospital stays, and postoperative hospitalization stays compared to Group B, with P-values < 0.001. The increased complexity of the “MESA-SVD” technique accounts for the extended surgical duration. Additionally, the difference in hospital stay durations is partly attributed to the clinical pathway of Group B, where most patients underwent preoperative assessments as outpatients and received day surgery upon admission.

As presented in Table 4, statistical analysis revealed no significant change in anastomotic inner diameter between 1 and 3 months postoperatively (*P* = 0.441), though blood flow velocity significantly increased (*P* = 0.032). By 6 months, both the inner diameter (*P* = 0.023) and blood flow velocity (*P* = 0.049) were significantly higher than those at 1 month. There were no significant differences between the 3-month and 6-month values (inner diameter: *P* = 0.13, blood flow velocity: *P* = 0.899), suggesting that anastomotic function had stabilized by 6 months postoperatively.

No anastomotic leakage or thrombosis was observed in any patient in Group A during follow-up. Furthermore, patients in this group showed sustained reductions in spermatic vein diameter and reflux time, indicating that the anastomosis maintained a stable and effective venous drainage pathway over time. These findings support the mid-term patency and physiological function of the reconstructed vessel.

As shown in Table 5, both groups A and B underwent semen analysis at 1, 3, and 6 months postoperatively.

There was no significant difference in sperm concentration between Group A and Group B at 1 month or 3 months postoperatively. However, by 6 months, Group A demonstrated a significant improvement in sperm concentration (36.88 ± 12.54 × 10^6/mL) compared to Group B (29.32 ± 10.34 × 10^6/mL, *P* = 0.024).

Progressive motility (PR) was consistently better in Group A compared to Group B across all time points. At 1 month, PR in Group A was 28.44 ± 7.90% versus 22.08 ± 9.91% in Group B (*P* = 0.003); this difference persisted at 3 months (35.16 ± 6.58% vs. 24.08 ± 12.27%, *P* < 0.001) and 6 months (38.24 ± 6.54% vs. 29.36 ± 10.60%, *P* < 0.001), indicating that MESA-SVD may support faster and sustained recovery of sperm motility.

Sperm morphology did not differ significantly between groups at 1 month. However, by 3 months, Group A showed significantly better morphology (2.98 ± 0.95% vs. 2.28 ± 1.02%, *P* = 0.016). This significant difference persisted at 6 months postoperatively (3.26 ± 0.46% vs. 2.66 ± 0.86%, *P* = 0.004), supporting the overall benefit of MESA-SVD in improving sperm morphology over time.

Overall, Group A exhibited significant improvements in sperm concentration, progressive motility, and sperm morphology—particularly between 3 and 6 months postoperatively—while Group B demonstrated only modest improvement in progressive motility, with no substantial gains in the other parameters.

In our study, although Group A (MESA-VSD) required a longer operative time and hospitalization than Group B (MSV), this combined approach achieved more substantial improvements in venous hemodynamics that translated to superior functional outcomes. The dual procedure not only addressed venous reflux through ligation but also established a physiological drainage pathway via the inferior epigastric shunt - a critical distinction that enabled sustained recovery of the testicular microenvironment.

The clinical significance of these hemodynamic improvements was particularly evident in semen parameters Group A exhibited a phased recovery pattern consistent with the spermatogenesis cycle: progressive improvements in motility and sperm morphology at three months, followed by increased sperm concentration at six months, aligning with the 72-day spermatogenic cycle and subsequent epididymal maturation. In contrast, Group B only showed marginal PR improvement by six months, with other parameters remaining suboptimal. These differential outcomes strongly suggest that the shunt procedure actively facilitates spermatogenic recovery rather than merely preventing further deterioration [26].

We hypothesize that the superior semen quality improvements in Group A resulted from effective drainage of the left renal vein via the newly established pathway into the inferior epigastric vein. This new drainage route likely prevents reflux around the testes, which may otherwise continuously impair spermatogenesis. Nonetheless, the percentage of morphologically normal sperm in Group A remained below 4% at six months, underscoring the need for continued follow-up to assess the potential for longer-term improvement.

The pathophysiological rationale for performing MSEA-SVD in patients with NCS-associated varicocele is to directly address the underlying left renal venous hypertension. In NCS, the compression of the LRV between the superior mesenteric artery and the aorta creates a proximal obstruction, leading to a significant pressure gradient between the LRV and the inferior vena cava. This hypertension is transmitted retrogradely into the left gonadal (spermatic) vein, which acts as a primary collateral pathway, resulting in venous dilation, valvular incompetence, and the clinical manifestation of varicocele [24]. Conventional varicocelectomy, such as MSV, merely ligates this collateral pathway. While it may alleviate the varicocele temporarily, it does not decompress the LRV and may theoretically exacerbate renal venous congestion by eliminating a major outflow route, potentially leading to symptom recurrence or the emergence of other collateral pathways.

Our MSEA-SVD procedure offers a hemodynamic solution by creating a new, low-resistance drainage route. The anastomosis between the proximal spermatic vein and the IEV effectively shunts the high-pressure blood from the LRV into the external iliac vein system via the IEV, bypassing the aortomesenteric compression site. This direct decompression of the LRV is evidenced by the significant postoperative reduction in the LRV peak velocity (PV1) and the pressure-velocity ratio (PVR) observed in our Group A patients (Table 8). By simultaneously performing selective venous disconnection (ligating other varicose veins while preserving lymphatics and the cremasteric artery), the procedure eliminates the pathological retrograde flow into the pampiniform plexus, thus treating the varicocele while simultaneously mitigating the root cause—renal vein hypertension. This dual approach of drainage and disconnection provides a more physiological correction of the aberrant hemodynamics in NCS.

The influence of varicocele on serum sex hormone levels remains controversial. Elevated venous pressure associated with varicocele can induce testicular ischemia and impair the function of Sertoli and Leydig cells, potentially disrupting hormone production and feedback mechanisms within the hypothalamic-pituitary-gonadal axis [27]. Despite this theoretical framework, our study did not observe significant postoperative changes in serum hormone or inhibin B levels in either group. This finding may reflect the unilateral nature of the condition and compensatory mechanisms of the contralateral testis. Future large-scale, longitudinal studies are needed to better understand the hormonal impact of varicocele and its surgical treatment.

From Table 7, in both groups, the left spermatic vein diameter and reflux duration significantly decreased at 1, 3, and 6 months postoperatively compared to baseline (*P* < 0.001 for all time points). Scrotal ultrasound follow-ups at six months showed no recurrence of varicocele in either group. Additionally, both groups exhibited significant reductions in spermatic vein diameter and reflux time, indicating effective surgical resolution. However, testicular asymmetry did not improve significantly, suggesting that recovery from varicocele-induced testicular atrophy may require a longer observation period.

According to Table 8, group A demonstrated a significant reduction in left renal vein blood flow velocity and PVR ratios, whereas Group B showed no notable changes postoperatively.

Postoperative ultrasound of the left renal vein revealed significant improvements in Group A, with notable reductions in PV1 and the PVR at 1, 3 and 6 months compared to both baseline and Group B (all *P* < 0.001). In contrast, Group B showed no notable changes postoperatively in PV1, PV2, or PVR over time. No significant differences were found in PV2 in either group throughout follow-up. Ultrasound assessment of the left renal vein revealed that Group A achieved effective relief of blood flow at the aortomesenteric angle, evidenced by a significant reduction in the pressure-to-velocity ratio (PVR). In contrast, Group B demonstrated no such improvement, with 11 patients exhibiting increased PV1 levels at six months postoperatively. This likely attributed to the increased flow resulting from the loss of the left gonadal vein/spermatic venous plexus as a collateral drainage pathway following its ligation. However, overall, no statistically significant difference in PV1 was observed between the 6-month postoperative and preoperative measurements. Therefore, whether microscopic varicocelectomy in patients with NCS and concomitant left VC leads to increased blood flow velocity in the left renal vein remains to be determined by larger-scale studies.

When contextualizing our findings within the existing literature on surgical management for NCS with varicocele, the MSEA-SVD technique occupies a distinct position. Compared to the robotic-assisted LRV transposition with distal gonadal vein anastomosis described by Xu et al. [28], which is a complex transperitoneal procedure requiring robotic infrastructure and addressing the compression via direct LRV repositioning, our MSEA-SVD technique is a minimally invasive, extraperitoneal microsurgical procedure. While the robotic technique effectively relieves the anatomic compression and establishes dual drainage, our approach focuses on creating an alternative physiologic drainage pathway without directly manipulating the aortomesenteric angle, resulting in shorter operative times and potentially lower morbidity.

Our technique aligns more closely with the microsurgical principle of spermatic-inferior epigastric vein anastomosis reported by Han et al. [29]. Both studies demonstrate the efficacy of this shunt in resolving symptoms and improving hemodynamic parameters. However, our study provides a direct comparative analysis against MSV in a randomized controlled trial design, offering higher-level evidence for the superior functional outcomes (semen quality) achieved with the shunt procedure. Furthermore, our detailed reporting of anastomotic patency and its evolution over time (Table 4) adds valuable data on the technical success and durability of the microvascular anastomosis. In contrast to endovascular stenting, which carries risks of migration and lifelong anticoagulation, particularly in young patients, and open surgical procedures with higher complication profiles, the MSEA-SVD technique presents a favorable risk-benefit ratio, effectively bridging the gap between simple ligation and more invasive reconstructive or endovascular options.

## Limitations of the Study

Limitations of this study include a relatively short follow-up period, small sample size, and single-center design, which restrict the generalizability of our findings. Future multicenter studies with larger patient cohorts and longer follow-up are necessary to validate these outcomes and further refine the surgical approach.

## Conclusion

Over a six-month follow-up period, Group A exhibited markedly better improvements in semen quality than Group B, likely due to reduced venous reflux and preservation of testicular function through the alternative drainage pathway. However, these benefits come with trade-offs: longer operative times, increased hospitalization and costs, the need for postoperative anticoagulation, along with potential complications such as anastomotic leakage or thrombosis.

The “Microscopic Ligation Combined with Shunt Procedure” demonstrates promising efficacy and safety for patients with nutcracker syndrome and concurrent left-sided varicocele. Despite requiring longer operative times, prolonged hospitalization, and higher costs compared to simple ligation, the procedure offers significant clinical advantages, including marked improvements in semen quality and effective renal vein decompression. These benefits suggest that the combined technique provides superior protections against venous reflux and testicular atrophy.

In summary, this study supports the safety and efficacy of the “Combined Anastomosis and Ligation” technique for treating NCS patients with concurrent VC. While larger, multicenter, and long-term studies are necessary, this technique has the potential to become the preferred surgical option due to its ability to simultaneously address spermatic vein dilation and renal vein compression.

## Data Availability

No datasets were generated or analysed during the current study.

## Abbreviations

ACEIs: Angiotensin-converting enzyme inhibitors
BMI: Body mass index
CTV: CT venography
FSH: Follicle-stimulating hormone
INH B: Inhibin B
LH: Luteinizing hormone
LVC: Left-sided varicocele
MSEA: Microsurgical Spermatico-Inferior Epigastric Vein Anastomosis
MSV: Microsurgical Varicocelectomy
NCS: Nutcracker Syndrome
PR: Progressive motility
PV1: The blood flow velocity at the compressed site of the left renal vein
PV2: The blood flow velocity at the widest part of the left renal vein near the renal hilum
PVR: The blood flow velocity ratio of the left renal vein, refers to the ratio of PV1 to PV2
SVD: Selective Venous Disconnection
T: Testosterone
